# Navigating the Digital Landscape for Potential Use of Mental Health Apps in Clinical Practice: Scoping Review

**DOI:** 10.2196/75640

**Published:** 2026-01-15

**Authors:** Nikki S Rickard, Perin Kurt, Tanya Meade

**Affiliations:** 1 Centre for Wellbeing Science Faculty of Education The University of Melbourne Carlton Australia; 2 School of Psychology Western Sydney University Sydney Australia

**Keywords:** American Psychological Association, anxiety, depression, digital mental health, functionality, mental health practitioner, mobile application, psychiatrist, psychologist, smartphones

## Abstract

**Background:**

The global demand for mental health services has significantly increased over the past decade, exacerbated by the COVID-19 pandemic. Digital resources, particularly smartphone apps, offer a flexible and scalable means of addressing the research-to-practice gap in mental health care. Clinicians play a crucial role in integrating these apps into mental health care, although practitioner-guided digital interventions have traditionally been considered more effective than stand-alone apps.

**Objective:**

This scoping review explored mental health practitioners’ views on potential use or integration of smartphone apps into clinical practice. We asked, “What is known about how mental health practitioners view the integration of smartphone apps into their practice?” Further, this scoping review explored the factors that might influence integration of smartphone apps into practice, such as practitioner and client characteristics, app design and functionality, and practitioner views.

**Methods:**

We conducted a systematic search of 3 databases that yielded 38 studies published between 2018 and 2025, involving 1894 participants across various mental health disciplines, most predominantly psychologists and psychiatrists. Data were collected on practitioner and client characteristics, app functionality, and factors deemed important or influencing practitioners’ opinions about app integration.

**Results:**

The included studies were most likely to explore use of apps outside the clinical session and focused on self-management apps for mental health monitoring and tracking, and for collecting data from the patient. Fewer studies explored use of apps within-session, or practitioner-guided apps. Practitioners prioritized app features aligned with the American Psychological Association’s evaluation criteria, with practitioners prioritizing engagement and interoperability, but also noted the importance of training and resourcing to support integration.

**Conclusions:**

While practitioners recognize the potential of apps in mental health care, integration into clinical practice remains limited. This study highlights the need for further research on practical implementation, clinical effectiveness, and practitioner training to facilitate the transition from potential to actual use of apps in mental health care settings. Recommendations include evaluating effectiveness of app integration through experimental studies and developing training modules to develop practitioners’ digital competencies and confidence in app use.

## Introduction

### Background

The global demand for mental health services has increased significantly over the past decade, with the World Health Organization reporting a 13% increase in the period from 2007-2017 [1]. The COVID-19 pandemic has placed an additional burden on the mental health system, with the prevalence of depression and anxiety disorders alone increasing by 25% [2]. Mental health professionals are struggling to meet this increased service demand, and there is a need for sustainable approaches to support professional care [3-5].

Digital resources offer a flexible, data-rich, and economical means of addressing the research-to-practice gap [6-9] and can be used to support the full spectrum of mental health care services [10-13]. Among the most rapidly increasing digital resources available in the mental health sphere are smartphone apps. With over 30,000 mental health smartphone apps (MHapps) available to the public, and smartphone ownership penetrating most sectors of the population, MHapps have the capacity to be an accessible and scalable adjunct to professional mental health care [14-19]. Smartphone apps can also offer quite sophisticated personalization for each user and are reported to be less stigmatizing for many than seeking professional care [17,18,20,21].

Clinicians are in a unique position to guide the integration of smartphone apps into mental health care. The views and capabilities of clinical staff are regarded as critical for successful integration of digital mental health tools [22]. The breadth of their influence is diverse and can include recommending reputable apps to clients to use independently, or using apps in- or out-of-session with ongoing support [23,24]. Digital interventions that are guided by a therapist or with in-person feedback are generally regarded to be more effective than self-management or “stand-alone” apps [16,25-29]. This is consistent with the widely accepted notion of a therapeutic alliance being a key mechanism underlying mental health treatment outcomes [30]. There is, however, some indication that a major benefit of guided digital interventions may be encouraging adherence to the intervention [25,31], and that the role of clinician could be reduced to key touchpoints such as onboarding at the end of every other module or on-demand [25]. While high-quality hybrid models such as the Digital Clinic [32,33] and Precision Behavioral Health [34] offer mobile app functionality (monitoring and interventions) fully integrated with strategically placed practitioner support (eg, through telehealth or in-person follow-ups), there is also a need to better understand how practitioners feel about integrating stand-alone apps into mental health care.

The capacity for smartphone app integration into mental health care also depends on the functionality of the app and the level of support it targets across the mental health spectrum. For example, app functions can include informing, recording, sharing data, reminding, communicating, and displaying [35,36]. Levels of support can range from lower (eg, well-being promotion, mood tracking, assessment, and psychoeducation) to higher (interventions and relapse prevention) intensity [24,37]. Digital mental health services are most confidently advocated as a low-intensity treatment within the stepped-care model, providing health promotion, prevention, and early intervention support for subclinical populations [11,17]. Digital apps are also generally claimed to be more effective for prevention and mild symptomatology [17,38], although some studies have observed stronger effects for individuals with more severe symptoms [39,40]. It would be of interest to better understand where mental health practitioners themselves consider app usage to be most acceptable.

Integration of smartphone apps into mental health care must therefore also consider what factors are important to the practitioner. In a survey of general health care providers, regulatory body support and an evidence base were ranked as the most important factors affecting practitioners’ decisions to incorporate digital tools into their practice [41]. Previous research has also suggested that remote monitoring tools are of particular interest to mental health providers [42]. Several evaluation frameworks describe key criteria identified as important to professional regulatory bodies like the American Psychological Association (APA). The APA’s criteria for evaluating MHapps include accessibility, privacy and security, evidence base, engagement, and interoperability [43,44]. While models such as the APA evaluation framework are critical for shaping regulation of digital tools like smartphone apps, the prioritization of standards may not necessarily align with what is important for individual practitioners. For example, while accessibility is the fundamental standard for MHapps, if practitioners are supplying devices or paying for the cost of the app, then this criterion may become less critical. In a recent evaluation of 100 of the most popular MHapps at the time [37], only one of those apps was found to meet all 5 standards expected by the APA evaluation model. The majority lacked basic accessibility, privacy, and security features, and only one met the final criterion of interoperability, which is important for integrating apps into broader mental health care.

### Rationale and Objectives

The use of smartphone apps among physicians and rehabilitation clinicians has previously been explored in other scoping reviews [45,46]; however, to date, no scoping reviews have been conducted on mental health practitioners’ consideration of use or integration of smartphone apps into clinical care. The affordances of smartphone apps may vary between different groups of professionals and in different clinical settings [45]. Accordingly, the aim of this scoping review was to examine research from the past 7 years to answer the broad research question, “What is known about how mental health practitioners view the integration of smartphone apps into their practice?” Further, this scoping review explored the factors that might influence integration of smartphone apps into practice such as practitioner and client characteristics, app design and functionality, and practitioner views.

## Methods

### Overview

A scoping review methodology was selected to identify and map key characteristics related to the uptake of smartphone apps by mental health practitioners [47]. This review was guided by the PRISMA-ScR (Preferred Reporting Items for Systematic reviews and Meta-Analysis extension for Scoping Reviews; Multimedia Appendix 1 [48]). The protocol was registered through the Open Science Framework [49].

### Search Strategy

A search was finalized in March 2025 using 3 electronic databases (PsycINFO, Web of Science, and IEEE Xplore). The search strategy was adapted for each database (Multimedia Appendix 2). Searches were conducted with a combination of terms related to mental health practitioners and smartphone MHapps. These terms were selected following a preliminary review of the literature. Searches were run against the title and abstract, and where possible, subject headings were combined with keywords. The search was restricted to peer-reviewed journal articles written in English and published between 2018 and March 2025.

### Eligibility Criteria

Selection of articles was based on the SPIDER (Sample, Phenomenon of Interest, Design, Evaluation, Research Type) framework [50], as shown in Table 1. This framework was chosen to capture qualitative, quantitative, and mixed methods studies.

**Table 1:** Eligibility criteria.

Category	Inclusion	Exclusion
Sample	Mental health practitioners, if more than 25% of the sample were psychologists, psychotherapists, psychiatrists, or other counsellors or social workers predominantly working in mental health	General practitioners, nurses, or other health workers not specific to mental health Studies that included both practitioners and other end users (eg, clients) but did not differentiate results by participant type
Phenomenon of Interest	Integration of MHapps^a^ into practice. Studies exploring the use, consideration, design, or trial of apps were included	Studies focusing on web-based mental health apps, as opposed to MHapps Studies focusing on only users’, rather than practitioners’, attitudes, behaviors, or experiences toward apps
Design	Interviews Surveys Observations Behavior measures	No original data collected
Evaluation	Attitudes, behaviors or experiences of mental health practitioners in consideration of, or use of, smartphone app integration into their practice	Studies focusing on only users’, rather than practitioners’, attitudes, behaviors, or experiences toward apps
Research Type	Qualitative Quantitative Mixed methods	Reviews and meta-analyses Commentaries Opinion pieces Study protocols

^a^MHapps: mental health smartphone apps.

### Selection

Searches were managed using EndNote X9 (Clarivate Analytics) and Microsoft Excel (Microsoft Corp). Duplicates were removed using the EndNote deduplication tool. One reviewer (PK) screened titles and abstracts to exclude obviously irrelevant articles and those identified as reviews, commentaries, or opinion pieces. The remaining titles and abstracts were then screened by 2 independent reviewers (NSR and PK). Disagreements were resolved by discussion, and where doubt existed, an independent assessment was conducted by a third reviewer (TM). Full texts were independently reviewed by all 3 authors. PK reviewed 100% of the articles, with NSR and TM each reviewing approximately 50% of the articles. Disagreements between each pair of reviewers were resolved by discussion to achieve consensus.

### Data Extraction and Analysis

An Excel spreadsheet was created to compile relevant data. Data items were sorted into 5 categories:

- Article information: including author, year of publication, country, aim and design.
- Practitioner characteristics: including sample size, type of practitioner (eg, psychologist), age, and gender.
- Client characteristics: including age, mental health condition (eg, depression), and population (eg, clinical).
- Characteristics of app use: including app name, app purpose (prevention/well-being promotion, psychoeducation, monitoring/tracking, assessment/case identification, treatment/intervention, continuing care/relapse prevention, other), app functionality according to the IMS Institute for Healthcare Informatics functionality score (IMS-11; inform, instruct, record [collects, shares, evaluates, intervenes], remind/alert, communicate, display, guide), patterns of use, in- and/or out-of-session, and practitioner guided and/or self-managed.
- Characteristics considered important and/or useful based on APA’s criteria: including accessibility, privacy and security, evidence base, engagement, interoperability, and other.

The data charting form was completed by 1 reviewer (PK), and a random subset (20%) of articles was independently verified for accuracy by NSR and TM. Studies were not critically appraised during this review.

### Data Synthesis

Study designs and methodologies were diverse, so data were synthesized in a descriptive format. Frequencies were determined where possible within variables, and trends were identified where appropriate in a narrative format.

## Results

### Selection of Sources of Evidence

**Figure 1:**
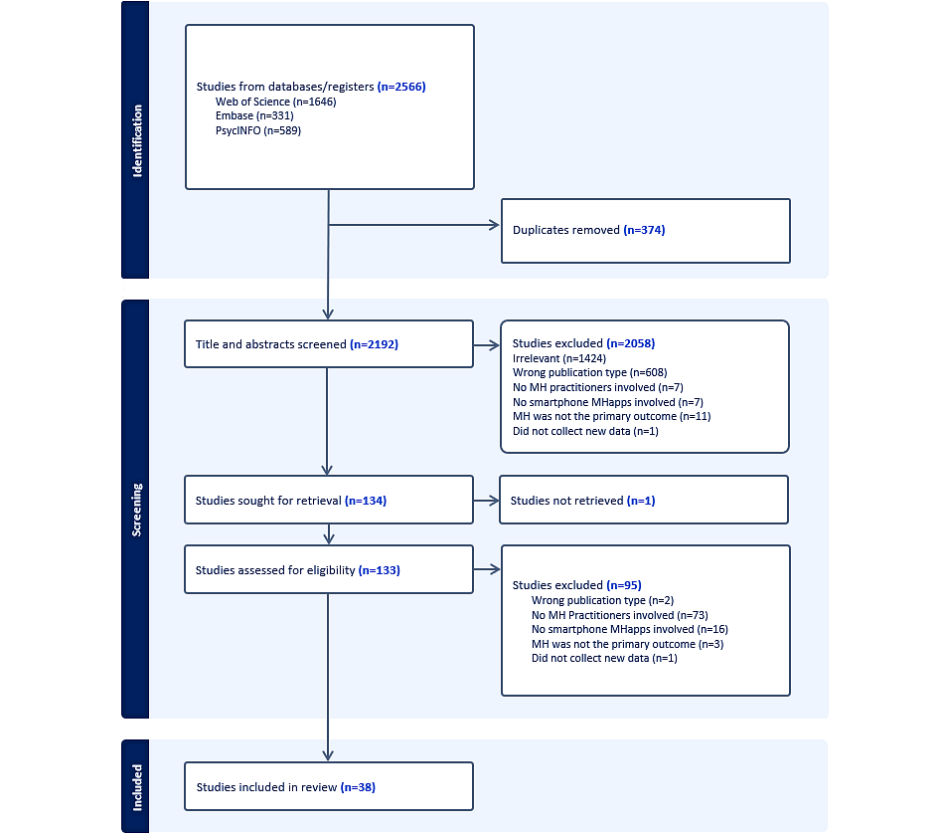
PRISMA (Preferred Reporting Items for Systematic Reviews and Meta-Analyses) 2020 flow diagram documenting literature search and outcome of screening process. MH: mental health; MHapps: mental health smartphone apps.

The initial search yielded 2566 records. After removing 374 duplicates, a total of 2192 titles and abstracts were screened, and 133 full-text publications were reviewed. Following full-text review, 38 publications were submitted to data extraction (Figure 1).

### Results of Individual Sources of Evidence and Synthesis of Results

The full characteristics of the included studies are outlined in Tables 2 and 3, with detailed information provided in Table S1 in Multimedia Appendix 3.

**Table 2:** Study and participant information of retrieved studies.

Author	Year	Country	Primary methodology	Sample size meeting criteria, n (% of N)	Practitioner age^a^ (years), mean (SD) or mode (SD)	Client age (years), mean (SD) or median (range)
Adams et al [51]	2021	US^b^	Mixed methods	11 (55% of 20)	Alpha: 43.3 (7.5) Beta 2: 37.8 (12.1)	Alpha: 15.3 (1) Beta: 15.9 (1)
Almadani et al [52]	2025	Saudi Arabia	Quantitative	135 (35% of 386)	<30 (61.1%) 30-40 (30.1%) 41-50 (06.0%) >50 (02.8%)	Not reported
Anastasiadou et al [53]	2019	Spain	Qualitative	7 (88% of 8)	34.63 (7.21)	15 (0.5)
Armstrong et al [54]	2021	US	Qualitative	6 (50% of 12)	38.42 (5.78)	18.9 (3.73)
Bucci et al [55]	2019	UK^c^	Qualitative	17 (35% of 48)	36.2 (SD not reported)	Not reported
Chang [56]	2023	US	Qualitative	5 (100% of 5)	Not reported	37 (10.7)
Cheung et al [57]	2023	Canada	Quantitative	98 (91% of 108)	Mode: 60+ (34.3%)	18-29 (20.5%) 30-39 (24.4%) 40-49 (20.6%) 50-59 (22.9%) 60+ (13%)
Deady et al [58]	2023	Australia	Mixed methods	3 (100% of 3)	Not reported	50 (range 42-56)
Dobson et al [59]	2022	New Zealand	Mixed methods	178 (91% of 195)	Mode: 24-35 (37%)	Not reported
Dominiak et al [60]	2024	Poland	Mixed methods	62 (42% of 148)	25-39 (27%) 40-55 (56%) 55-64 (15%)	Not reported
Dubad et al [61]	2021	UK	Mixed methods	3 (50% of 6)	Not reported	20.71 (2.56)
Etingen et al [62]	2024	US	Mixed methods	230 (81% of 284)	Under 35 (19.2%) 36-45 (50.5%) 46-55 (19.2%) 56-65 (9.3%) 66-75 (1.6%)	Not reported
Francese et al [63]	2023	Italy	Quantitative	8 (100% of 8)	43 (6.18)	36.24 (14.02)
Gonzalez-Perez et al [64]	2024	Spain	Mixed methods	9 (100% of 9)	35 (SD not reported)	Not reported
Green et al [65]	2023	US	Mixed methods	7 (78% of 9)	Not reported	19.6 (2.05)
Heydarian et al [66]	2023	Iran	Mixed methods	7 (54% of 13)	Not reported	Not reported
Hildebrand et al [67]	2024	Germany	Qualitative	269 (77% of 350)	42.83 (12.16)	Not reported
Hoffman et al [68]	2019	US	Mixed methods	15 (63% of 24)	Not reported	36.5
Kerst et al [69]	2020	Germany	Quantitative	33 (58% of 57)	43 (12.3)	Not reported
Khan et al [70]	2023	Canada	Qualitative	2 (33% of 6)	Range: 32-48	Range: 32-48
Li et al [71]	2022	Australia	Quantitative	10 (31% of 32)	38.7 (10.4)	14.94 (1.3)
Lukka et al [72]	2023	Finland	Qualitative	11 (61% of 19)	18-29 (5%) 30-39 (16%) 40-49 (32%) 50-59 (37%) 60-69 (11%)	Not reported
McGee-Vincent et al [73]	2023	US	Quantitative	271 (25% of 1107)	MHSL staff (44.2, 10.2) AOSL staff (46.0, 10.9)	Not reported
Medich et al [74]	2023	US	Qualitative	10 (63% of 16)	Not reported	Median: 45, Range: 21-66 years
Miller et al [75]	2019	US	Mixed methods	103 (47% of 220)	45.5 (11.1)	Not reported
Morton et al [76]	2021	Multiple	Mixed methods	57 (71% of 80)	44.7 (13.1)	Not reported
Naccache et al [77]	2021	France	Mixed methods	3 (43% of 7)	36.7 (7.38)	15.5 (1.07)
Nogueira-Leite et al [78]	2023	Portugal	Mixed methods	152 (95% of 160)	<26 (2.5%) 26-35 (35%) 36-45 (36.9%) 46-55 (19.4%) 56-65 (3.8%) >65 (2.5%)	Not reported
Orengo-Aguayo et al [79]	2018	US	Mixed methods	7 (78% of 9; Phase 1) and 35 (63% of 56; Phase 2)	Phase 1: 41.88 (10.25) Phase 2: 39.73 (10.26)	Not reported (although we know that the clinicians treated children and adolescents)
Patoz et al [80]	2021	France	Qualitative	16 (62% of 26)	45.5 (12.2)	51.5 (15.5)
Puhy et al [81]	2021	US	Mixed methods	3 (100% of 3)	Not reported	16.1 (1.2)
Richards et al [82]	2018	Australia	Qualitative	6 (100% of 6)	Not reported	Not reported (although we know that the patients were 18 years and older)
Rodriguez-Villa et al [83]	2021	Multiple	Qualitative	35 (67% of 52)	BIDMC^d^: 41 (SD not reported) AIIMS^e^: 35 (SD not reported) NIMHANS^f^: 41 (SD not reported)	BIDMC: 32 (SD not reported) AIIMS: 33 (SD not reported) NIMHANS: 35 (SD not reported)
Rothmann et al [84]	2022	Denmark	Qualitative	3 (100% of 3)	Not reported	Only reported range = 25-59 years
Stefancic et al [85]	2022	US	Qualitative	8 (73% of 11)	Not reported	Not reported (although we know the clinics provide treatment to adolescents and young adults aged 16-30 years)
Strodl et al [86]	2020	Australia	Mixed methods	38 (60% of 63)	Focus group: 47 (10.5) Telephone sample: 45.7 (10.5)	Not reported
Weermeijer et al [87]	2023	Belgium	Mixed methods	9 (75% of 12)	Users: 45.57 (6.11) Dropouts or nonusers: 43.50 (17.50)	Users: 34.93 (11.27) Dropouts or nonusers: 36.67 (13.47)
Wu et al [88]	2020	US	Mixed methods	12 (100% of 12)	Not reported	Not reported

^a^Mean age and gender typically reported for all practitioners, and sometimes all of sample.

^b^US: United States.

^c^UK: United Kingdom.

^d^BIDMC: Beth Israel Deaconess Medical Center.

^e^AIIMS: All India Institute of Medical Sciences.

^f^NIMHANS: National Institute of Mental Health and Neurosciences.

**Table 3:** Characteristics of app use in retrieved studies, and American Psychiatric Association (APA) standards prioritized by practitioners.

Author	Use of app for research or practice	App purpose	In-session and/or out-of-session	Practitioner guided and/or self-managed	APA standards prioritized
Adams et al [51]	Research	Prevention/promotion Assessment/case identification	Both	Both	A^a^, SP^b^, EB^c^, E^d^, I^e^
Almadani et al [52]	Clinical	Psychoeducation monitoring/tracking Treatment/intervention	Not reported	Not reported	A, SP, EB
Anastasiadou et al [53]	Research	Monitoring/tracking Continuing care/relapse prevention	Out-of-session	Practitioner guided	A, SP, EB, E, I
Armstrong et al [54]	Research	Monitoring/tracking	Out-of-session	Not reported	A, SP, EB, E, I
Bucci et al [55]	Research	Monitoring/tracking Treatment/intervention	Out-of-session	Self-managed	A, SP, E, I
Chang et al [56]	Clinical	Monitoring/tracking	Out-of-session	Not reported	A, SP, EB
Cheung et al [57]	Clinical	Assessment/case identification Monitoring/tracking	Not reported	Not reported	A, SP
Deady et al [58]	Research	Psychoeducation Treatment/intervention	Out-of-session	Practitioner guided	A, EB, E, I
Dobson et al [59]	Clinical	Not reported	Not reported	Not reported	SP
Dominiak et al [60]	Clinical	Prevention/well-being promotion Monitoring/tracking Continuing care/relapse prevention	Not reported	Not reported	A, EB, E, I
Dubad et al [61]	Research	Monitoring/tracking	Not reported	Not reported	I
Etingen et al [62]	Research	Monitoring/tracking Assessment/case identification	Out-of-session	Practitioner guided	E, I
Francese et al [63]	Research	Assessment/case identification Monitoring/tracking	In-session	Practitioner guided	E, I
Gonzalez-Perez et al [64]	Research	Psychoeducation Monitoring/tracking Assessment/case identification Treatment/intervention	Out-of-session	Self-managed	Not available
Green et al [65]	Clinical	Psychoeducation Monitoring/tracking Treatment/intervention	Out-of-session	Practitioner guided	A, EB, E, I
Heydarian et al [66]	Research	Psychoeducation Monitoring/tracking	Not reported	Self-managed	SP, EB, I
Hildebrand et al [67]	Clinical	Psychoeducation Treatment/intervention	Out-of-session	Not reported	A, EB, E
Hoffman et al [68]	Clinical	Treatment/intervention Psychoeducation Monitoring/tracking	Out-of-session	Self-managed	A, SP, EB, E, I
Kerst et al [69]	Clinical	Treatment/intervention	Not reported	Not reported	A, SP, I
Khan et al [70]	Clinical	Psychoeducation Monitoring/tracking Treatment/intervention	Not reported	Not reported	SP, E, I
Li et al [71]	Research	Treatment/intervention (cognitive behavioral therapy) Psychoeducation (activities for depression)	Out-of-session	Self-managed	A, SP, EB, E, I
Lukka et al [72]	Clinical	Psychoeducation Treatment/intervention	Not reported	Both	SP, EB, E
McGee-Vincent et al [73]	Clinical	Not reported	Not reported	Not reported	A, SP, EB, E
Medich et al [74]	Research	Monitoring/tracking	Out-of-session	Self-managed	EB, E
Miller et al [75]	Clinical	Monitoring/tracking	Out-of-session	Self-managed	A, SP, EB, E, I
Morton et al [76]	Clinical	Monitoring/tracking Treatment/intervention	Not reported	Self-managed	A, SP, EB, E, I
Naccache et al [77]	Research	Prevention/promotion Monitoring/tracking Treatment/intervention	Not reported	Self-managed	A, SP, EB, E, I
Nogueira-Leite et al [78]	Clinical	Monitoring/tracking	Not reported	Not reported	A, EB, E, I
Orengo-Aguayo et al [79]	Research	Prevention/promotion Monitoring/tracking Assessment/case identification	Both	Practitioner guided	A, SP, EB, E, I
Patoz et al [80]	Research	Prevention/promotion Monitoring/tracking Assessment/case identification	Out-of-session	Self-managed	A, SP, EB, E, I
Puhy et al [81]	Research	Prevention/promotion Monitoring/tracking	Both	Both	SP, EB, E, I
Richards et al [82]	Research	Tracking/monitoring Assessment/case identification	Out-of-session	Both	A, E, I
Rodriguez-Villa et al [83]	Research	Continuing care/relapse prevention	Both	Self-managed	A, SP, EB, I
Rothmann et al [84]	Research	Prevention/promotion Treatment/intervention	Both	Practitioner guided	A, E
Stefancic et al [85]	Research	Assessment/case identification	Out-of-session	Self-managed	A, SP, EB, E, I
Strodl et al [86]	Research	Prevention/promotion Tracking/monitoring Assessment/case identification	Out-of-session	Both	A, SP, EB, E, I
Weermeijer et al [87]	Research	Monitoring/tracking	In-session	Not reported	A, EB, E
Wu et al [88]	Clinical	Monitoring/tracking	Out-of-session	Practitioner guided	EB, E, I

^a^A: accessibility.

^b^SP: security and privacy.

^c^EB: evidence base.

^d^E: engagement.

^e^I: interoperability.

### Studies’ Characteristics

Publications were distributed evenly across the 8-year period of inclusion (2018-2025), with a peak in 2023 during which 32% (12/38) of included studies were published (Table 2). The largest representation of studies was from the United States (12/38, 32% of studies), followed by Australia (4/38, 11% of studies), with only one or two studies across each of the remaining 14 countries. The total number of practitioners sampled within the retrieved studies was 1894, with sample sizes ranging from 3 [84] to 271 [73], and 11 being the median sample size.

The studies’ aims (Table S1 in Multimedia Appendix 3) covered a wide range of topics related to the use of mobile apps in mental health care, with about half of the retrieved studies reporting on clinicians’ attitudes, experiences, and their views on acceptability and feasibility of using digital tools in mental health care. Usability studies involved developing and testing MHapps for a variety of conditions, with depression the most common, followed by general mental health and multiple disorders. Less commonly, studies focused on evaluating specific features of mental health apps, such as mood monitoring components, or on developing training programs for health care professionals and assessing their digital competence. The studies used a diverse range of methodological approaches, with the majority using qualitative or mixed methodologies to explore the perspectives, experiences, and needs of stakeholders regarding the use of mobile apps in mental health care. More than half the studies (20/38, 53% of total) were mixed methods designs, while 12 studies (32% of total) were qualitative designs (involving for example, focus groups, semistructured interviews). Only 6 of the 38 (16%) studies had an entirely quantitative design (involving cross-sectional and longitudinal surveys or questionnaires). Several studies focused in designing new apps and included co-design or user-centered design approaches, which emphasized the involvement of end-users (practitioners, clients, or both) in the development and refinement of mobile apps.

### Practitioner and Client Information

A range of mental health practitioners were represented in the included studies (Table S1 in Multimedia Appendix 3), with psychologists the most common (in 32 studies) and psychiatrists the next most common (in 21 studies). Practitioner age varied significantly across studies, with modal age ranging from <30 years to >60 years, and there were more female (40.7% to 100% range) than male or nonbinary practitioners represented. Clients’ age ranged from 15-52 years, with the most common age range (when reported) being adolescents and young adults (8 of the 38 studies). The majority of studies (29 of 38 studies) appeared to include clinical samples, although reporting was at times ambiguous. The most commonly reported mental health conditions in the client sample were mood disorders (depression, anxiety, and bipolar disorder), following by general mental health conditions and multiple disorders.

### App Characteristics

**Figure 2:**
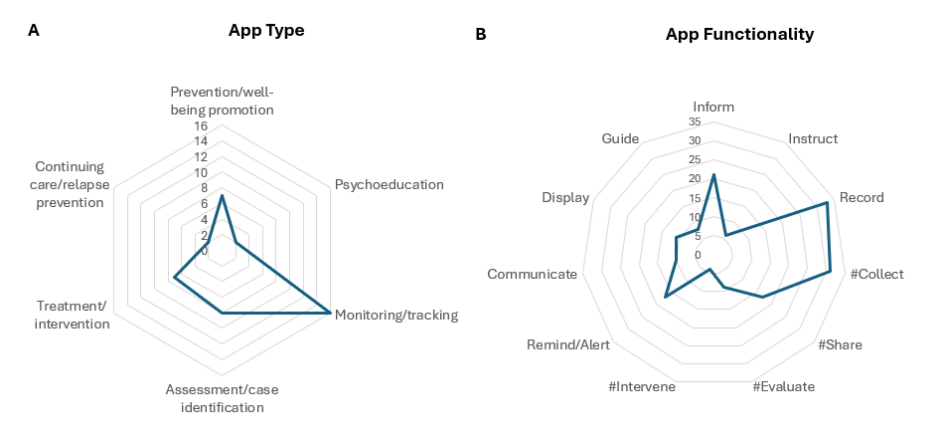
Radar plots of (A) purpose of apps and (B) app functionality (IMS Institute for Healthcare Informatics functionality score [IMS-11]). Categories prefixed with # represent the 4 subfunctions of the recording function.

Less than half of the included studies (16/38, 42%) involved practitioners’ use of apps integrated into their routine clinical practice, rather than as part of trials or feasibility research studies (Table 3). Almost half of the studies (18/38, 47%) explored the use of a specific app, with 4 (11%) studies examining a toolkit of apps (Table S1 in Multimedia Appendix 3). The remaining studies explored practitioners’ attitudes or use of mental health apps more generally. The majority of apps discussed across studies were monitoring/tracking apps, followed by treatment/intervention apps, psychoeducation, and assessment/case identification apps (Table 3 and Figure 2A). With regard to app functionality (Figure 2B), the majority (25/38, 66%) of studies cited apps which had 3 or fewer of the 7 IMS-11 app functions. Most studies (33/38, 87%) reported on apps that included a recording function, with nearly all studies (31/38, 82%) reporting on apps that collected data from users. The next most common function reported in studies was informing (21/38, 55%), followed by reminding/alerting and recording for the purpose of sharing data (each reported in 17/38, 45% of studies). The least common functions were instructing (reported in only 6/38, 16% of studies) and intervening (reported in 4/38, 11% of studies).

Apps were used primarily out-of-session (18/38, 47% of studies), with 5 studies (13%) citing app use both in- and out-of-session. Only 2 studies [63,87] focused on an app used specifically in clinical sessions. The most common management approach reported was self-management (12/38, 32% of studies), with 5 studies (13%) reporting both practitioner-guided and self-management, and 8 studies (21%) reporting practitioner-guided only. The remainder of studies did not report the management approach.

A total of 16 of the 38 (42%) retrieved studies indicated practitioners’ current patterns of using apps with their clients. In the majority of these studies (31/38, 82%), practitioners had incorporated apps into their practice by either using them or recommending them to clients. Patterns of use included providing a list of apps to patients to explore, or recommending a specific app with varying levels of instructions or follow up. Where described, practitioner’s intentions tended to be for patients to self-manage their health [68,75,76,83], with follow-up or review rarely reported. In other studies, practitioners intended to use apps in the future for their patients [52,78]. In one study, the likelihood of prescribing apps increased following practitioner training designed to increase the reach of MHapps for veterans [73].

### Practitioners’ Prioritization of App Features Mapped Onto APA App Quality Criteria

All 5 APA criteria were recognized as important across the majority of studies (Table 3). The criterion most commonly raised was engagement, cited by practitioners in 79% (30/38) of retrieved studies. Factors of importance to practitioners included ease of use and user-friendliness, attractive and intuitive design, ability to personalize and customize features, inclusion of reminders and notifications, gamification and interactive elements, and provision of feedback and progress tracking. For example, Li et al [71] emphasized the importance of incorporating interaction patterns and functionalities used in popular social media apps, such as swipe interactions and short videos, to enhance engagement for a youth-focused app.

Similar in importance to practitioners was Interoperability, cited in 76% (29/38) of studies, with key factors including the ability to share data with clinicians or caregivers, integration with electronic health records, supporting in-session and between-session use, and compatibility with clinician workflows and systems. For example, Wu et al [88] highlighted the importance of apps that seamlessly integrate data into electronic health records and support collaborative care, suggesting the value of interoperability for practitioners.

Accessibility and evidence base were also commonly cited criteria by practitioners (cited in 26/38, 68% and 27/38, 71% of the studies respectively). Accessibility included factors associated with smartphone ownership (due to technical resources such as Wi-Fi or cost), availability on multiple platforms, language requirements, ability to use offline or on demand, and data storage requirements. Regarding the importance of an evidence base, practitioners noted the importance of including credible, reliable, and up-to-date content; the use of validated scales and questionnaires; alignment with clinical practice guidelines; provision of psychoeducation; information being appropriate for specific mental health conditions; and information about the underlying science and research. For example, Miller et al [75] noted that the intention to use apps in the future was influenced by professional training, followed by scientific evidence, suggesting the importance of an evidence base for practitioners. Security and privacy concerns were the least frequently cited issue by practitioners although still cited in 63% (24/ 38) of studies. Concerns included data privacy and confidentiality, security protocols (eg, passwords, encryption), regulations and liability governing data access and use, and ensuring user privacy when sharing information with clinicians or caregivers. For example, Anastasiadou et al [53] highlighted concerns about a restrictive health care system in Spain that limited the sharing of patient information online, emphasizing the importance of addressing privacy and security concerns. Given all 5 APA criteria were raised as important across most studies, it was not possible to discern any patterns or associations between APA criteria raised and client or practitioner characteristics.

Other issues identified as important by practitioners beyond the formal APA app quality evaluation model included a range of implementation factors, such as the importance of clinician training in using the technology to enhance familiarity and confidence in using and recommending apps, and organizational investment in resources and support for integrating digital mental health into practice. Client factors were also noted, including the potential impact of apps on therapeutic alliance and treatment outcomes, and moderation of app utility by client characteristics, including digital literacy, age, and motivation.

## Discussion

### Overview

The aim of this scoping review was to explore what is known about the mental health practitioners’ views on integration of smartphone apps into their practice; the factors that may influence such integration, including practitioner and client characteristics, app functionality and design; and reported use of those apps in practice.

### Main Findings

Across the 38 included studies, a total of 1894 mental health professionals participated, with sample sizes ranging from 3 to 271. The participants predominantly included psychologists and psychiatrists, as well as a number of other mental health practitioners, with participants being predominantly female and mid-career based on their age range (30-39 years and 40-49 years). Practitioners worked across a range of services, from specialized clinics (eg, eating disorders and psychosis early intervention) to school- or tertiary-based counselling units, demonstrating a diverse and representative sample of mental health practitioners and services. Notably, however, more than half (24 of the 38) of the studies had sample sizes less than 50, and some demographic features such as age or gender, were not reported by all studies, which limits the degree of generalizability of views to a broader mental health practitioner population.

From the demographic information included, the participants appear to be predominantly experienced and qualified practitioners actively involved in mental health services. Practitioners’ clients across the included studies ranged from children to adolescents and adults, predominantly from clinical settings and presenting with a range of mental health issues, most commonly mood and anxiety disorders. This is consistent with the prevalence of those mental health conditions in both clinical and community populations, which have further increased post the COVID-19 pandemic [2]. The increased prevalence of those common conditions continues to challenge limited health services, access and continuity of care and practitioners’ ability to meet the needs of growing number of clients. The consistency in the mental health conditions in the population, and those identified in this review of practitioners who are exploring smartphone apps in their practice, is therefore promising for alignment of support demand and practitioner readiness for integration of digital tools into their practice. Therefore, smartphone apps offer a viable add-on resource and a self-directed mental health support.

However, much of the current literature on the use of smartphone apps in mental health care is predominantly focused on the development of such apps and exploration of perspectives of both practitioners and clients on their usability rather than on actual usage patterns or clinical effectiveness. This aligns with recognition of the importance of co-designing apps to engage end users (which can be both practitioners and clients), and better understand their needs and preferences [22]. Across the 38 studies, about half reported app use out-of-sessions, or in- and out-of-session, with only 2 out of 38 reporting in-session use only. It therefore appears that while the use of apps is recognized by practitioners as potentially helpful to their clients, the actual use remains consistent with stepped-care models of mental health care, in which digital technologies are regarded as best suited for self-managed, low-intensity support [12,89] rather than as part of an integrated in-session care plan. This is understandable, given that the primary focus of the apps included across those studies was on well-being promotion, tracking/monitoring, psychoeducation, early intervention, and assessment monitoring, as well as their capacity to collect, record, and inform. In contrast, about one-third of studies reported using apps within the higher-intensity range of treatment/intervention or continued care/relapse prevention functions, with few apps offering “instructing” or “intervening” functions. Interestingly, however, the proportion of apps offering these higher-intensity support options appears to be increasing, with the proportion doubling from 17% to 34% in the final year covered by this review. Most apps remain limited in the scope of their functionality, offering 3 or fewer functions. Apps that offer a greater multifunctionality in their design are more likely to be useful to client-practitioner sessions [36], suggesting a need for broader spectrum, “all-in-one” apps.

Despite previous literature suggesting practitioner-guided apps are more effective than self-managed or “stand-alone” apps [16,25), the studies identified in this review focused more on self-management apps. The majority of studies (17/38, 45%) included self-management apps, with 32% (12/38) entirely stand-alone. While there is currently still limited evidence of effectiveness for stand-alone apps [16], this delivery mode may be of greater interest to practitioners given the persistent high demand for their services [90]. Self-management apps also align with models of health care that aim to empower patients to participate more in the management of their own health [91]. This is despite some reports that involvement of practitioners in client use of mental health apps aids effectiveness of those apps in comparison to self-managed or stand-alone app use [26,27]. There is, however, a need for research to capture what that involvement looks like and to what degree it facilitates the effectiveness of the app and its contribution to the clinical sessions. Practitioners are more likely to recommend apps (whether used in- or out-of-session) if there is clear evidence of how their involvement and the client’s use of the app contributes to the clinical care outcomes.

Given that the majority of mental health apps do not meet all 5 standards outlined by the APA evaluation model [37], it is understandable that their use by mental health practitioners is still at a potential rather than actual and evidence-based stage. Notably, across the 38 studies reviewed, practitioners raised the importance of app features consistent with those 5 APA standards, indicating an informed position in their considerations of suitability of apps for integration into mental health care. It is notable that engagement and interoperability were most commonly identified by practitioners as important. Practitioners’ awareness of the need for an engaging user experience is promising, given its critical role in compliance and sustainability of use [9,92,93]. Similarly, the importance of data sharing is consistent with digital tools being used as an adjunct rather than alternative to professional care, and providing practitioners with information that may contribute to their in-session interactions with the client [44,94]. Integration of assessment and monitoring data into electronic records may also provide new insights which may not be possible with more manual recording of less regular data points. It is also promising that an evidence or clinical base for apps was reported in 71% (27/38) of studies. This reflects an awareness that mental health apps need to be credible and developed by trusted sources, and for the function and outcomes of the app to be aligned through systematic evaluation. In this context, it is of concern that the majority of mental health apps available publicly lack sufficient evidence or clinical support for their effectiveness [15,95].

Security and privacy and accessibility were each also reported across the majority of studies. The importance placed on security and privacy demonstrates practitioners’ awareness of the importance of ethical management of digital patient records [96]. The equally high importance of accessibility reflects practitioners’ awareness that mental health apps must be designed and distributed in a way that does not exclude any sociodemographic groups, and encourages uptake and sustained use for all. Among the other criteria raised by practitioners beyond the APA model, the need for organizational support in training and technical resource support was a commonly reported concern. Implementation of training modules has been effectively demonstrated to enhance knowledge and confidence of mental health practitioners about using apps [73]. However, training on digital mental health options for practitioners remains limited [97]. These are important practical considerations in facilitating progression from potential to actual use of apps in clinical settings and should be considered for all mental health staff working within clinical settings.

To progress the integration of mental health apps into clinical sessions, it may be informative to understand the context of clinical sessions that include assessment, formulation, treatment plan, monitoring of progress, and self-directed tasks between sessions. In addition, it would be helpful to include information on which APA standards a mental health app meets in app stores, or some other mental health register, to assist selection for a particular client with a particular mental health condition. Without a closer alignment between what may aid sessions and a client’s in- and out-of-session support, self-development- and psychoeducation, there may be too many apps for practitioners to choose from without a clear sense of how useful those apps may be or how to measure their add-on effectiveness.

Overall, the 38 studies included in this review provide an informative understanding of what practitioners consider important in the design and function of mental health apps if they were to use or integrate their use into their clinical sessions. Further research is required to capture what that use may look like in practice and whether using particular apps provides additional benefit to the clinical session and the therapeutic output, or provides support in between sessions that may otherwise be lacking. Given the constrains of limited mental health services, anything that may be added to the sessions’ content (ie, review of app-collected data) or between sessions (ie, client and practitioner’s engagement with the app) must have a clear add-on clinical value.

### Limitations

Limitations across studies include small sample sizes, limited demographic and descriptive information, overrepresentation of studies from the United States and early adopters of integration of apps into clinical practice, which means that the findings across those studies may not be generalizable to broader populations of practitioners. Further, the studies included in this scoping review were inclusive of studies exploring both views of potential and actual integration of apps into practice and therefore cannot provide indication of prevalence of use. While some of the studies have provided indication of factors that may influence integration, or not, of apps into clinical practice, this is an area that requires further research across broader practitioners’ representation. Further, real-world factors relating to successful integration of apps into practice—such as patient acceptability, organizational culture around adoption, and resourcing and feasibility of sustained use—were not within the scope of this review [22,98].

This review also focused on mental health professionals only to contain the scope of practice. A similar review could be undertaken with other health professionals who work with mental health issues in a different therapeutic context (eg, general practitioners, nurses, and other allied health practitioners), which may reveal different uses of available apps. The quality of included studies was also not assessed, as this was a scoping, not a systematic, review and therefore relied on descriptive rather than evaluative presentation of those studies.

### Future Directions and Recommendations

Notwithstanding these limitations, the 38 studies offer an overview of several useful mental health apps that could be integrated into clinical practice, ranging from between-session monitoring/tracking data to interactive relapse prevention apps that may provide useful between-session engagement and within-session progress-informing data. This scoping review has identified that practitioners are interested in the use of low-intensity support apps outside of sessions, which is aligned with the stepped-care model and 2 key points: addressing support gaps between sessions, which are critical to retention and relapse prevention; and empowering clients to self-manage some aspects of their health.

As a way forward, it is recommended that the potential use of some apps be explored in a clinical setting using well-structured experimental designs to assess suitability, usability, and effectiveness of those apps beyond their potential and ad hoc use. A good starting point may be integrating the use of apps outside of sessions, with a review of their use, clients’ perceptions of usefulness, and, where relevant, review of data during sessions, similar to the common practice of giving clients homework (eg, new skills to practice, daily mood-monitor sheets, or behavior tracking).

For practitioners who would like to use mental health apps, it is recommended that they base their selection of apps on the APA standards as a starting point, focusing on apps that align with particular aspects of a client’s care plan (eg, relaxation for anxiety or daily pleasant activities for mood), and then determine how they may like to integrate them in their clinic sessions (eg, for psychoeducation or monitoring symptoms). Some practitioners may find reviewing apps against the APA standards relatively straightforward, especially if they are comfortable users of digital technology. Others, however, may require some support, given that there is a large variation in digital competencies, with many having low awareness and knowledge [59,68,69] and confidence in suggesting apps to their clients [76]. It is therefore recommended that educational training modules or workshops are developed to inform use of mental health apps in practice, starting with low-intensity app selection for use between session to high-intensity apps for within sessions for more advanced digital technology-user practitioners. Such training needs to address commonly raised concerns by both practitioners and clients, including accessibility, safety, and potential side effects [80], and build practitioners’ self-efficacy in the use of apps with their clients [76]. An implementation science lens [22,99] would be useful to extend this review into consideration of factors that support successful integration of mental health apps into practice.

### Conclusion

This scoping review has provided valuable insights into mental health practitioners’ perspectives on integrating smartphone apps into their practice. The findings reveal that practitioners are interested in the capabilities of these digital tools, particularly for self-management and out-of-session support, aligning with stepped-care models of mental health care. The use of mental health apps is an untapped resource that can support both practitioners and clients in improving engagement and implementation of care plan strategies [86] and a reciprocal responsibility for mental health management [82]. This may be particularly relevant for practitioners who are regular uses of digital technologies and who can be at the forefront of apps integration into practice, as well as practitioners whose clients are highly engaged with technology as a health self-management resource. However, the actual integration of apps into clinical practice remains limited, with practitioners emphasizing the importance of app features such as interoperability, security, privacy, engagement, and accessibility. This review highlights a gap between consideration and actual use of mental health apps in clinical settings, suggesting a need for further research to explore the practical implementation and effectiveness of these tools in therapeutic contexts. To facilitate the adoption of mental health apps in clinical practice, it is recommended that:

- Practitioners base their app selection on established standards, such as those outlined by the APA.
- Educational training modules or workshops be developed to enhance practitioners’ digital competencies and confidence in using apps with clients.
- Well-structured experimental designs be used to assess the suitability, usability, and effectiveness of apps in clinical settings.
- App developers and researchers work toward aligning app functionalities more fully with a range of therapeutic needs and contexts.

As the field of digital mental health continues to evolve, ongoing collaboration between practitioners, researchers, and app developers will be crucial in realizing the full potential of smartphone apps as valuable adjuncts to traditional mental health care.

## Appendix

Multimedia Appendix 1 PRISMA-ScR checklist.

Multimedia Appendix 2 Search strategy detail.

Multimedia Appendix 3 Additional characteristics of studies, sample and apps in retrieved studies.

## Abbreviations

APA: American Psychiatric Association
IMS-11: IMS Institute for Healthcare Informatics functionality score
MHapps: mental health smartphone apps
PRISMA-ScR: Preferred Reporting Items for Systematic reviews and Meta-Analysis extension for Scoping Reviews
SPIDER: Sample, Phenomenon of Interest, Design, Evaluation, Research Type
