# Supplementary material for: Navigating the Digital Landscape for Potential Use of Mental Health Apps in Clinical Practice: Scoping Review
Source: JMIR Ment Health. 2026 Jan 15;13:e75640. doi: 10.2196/75640 (PMC12856407; doi:10.2196/75640)
Supplement: Multimedia Appendix 2 [file mental_v13i1e75640_app2.docx]

**Multimedia Appendix 2**

**PsycINFO – Subject Headings, TI, AB**

*Mental Health Personnel (Explode), Psychologists, Counseling Psychologist, Counselors, Clinicians, Physician*

psychologist OR psychotherapist OR psychiatrist OR counsellor OR counselor OR clinician OR practitioner OR physician OR “mental health provider*” OR “mental health worker*” OR “mental health professional*” OR “mental health personnel” OR “mental health staff” OR “healthcare provider*” OR “healthcare worker*” “healthcare professional*” OR “healthcare personnel” OR “healthcare staff” OR “clinic* staff” OR “clinic* personnel”

*Mobile Applications*

app OR application OR apps OR applications

*Smartphones, Mobile Phones*

mobile OR smartphone

*Mental Health, Mobile Health*

“mental health” OR “e-mental health” OR “electronic mental health” OR “digital mental health” OR psych* OR wellbeing OR “well being” OR wellness OR depression OR anxiety OR mood

**Web of Science – TI, AB**

psychologist* OR psychotherapist* OR psychiatrist* OR counsellor* OR counselor* OR clinician* OR practitioner* OR physician* OR “mental health provider*” OR “mental health worker*” OR “mental health professional*” OR “mental health personnel” OR “mental health staff” OR “healthcare provider*” OR “healthcare worker*” “healthcare professional*” OR “healthcare personnel” OR “healthcare staff” OR “clinic* staff” OR “clinic* personnel”

app OR application OR apps OR applications

mobile OR smartphone

“mental health” OR “e-mental health” OR “electronic mental health” OR “digital mental health” OR psych* OR wellbeing OR “well being” OR wellness OR depression OR anxiety OR mood

**IEEE Xplore – All Metadata**

psychologist OR psychotherapist OR psychiatrist OR counsellor OR clinician OR practitioner OR physician OR “mental health provider*” OR “mental health worker*” OR “mental health professional*” OR “mental health personnel” OR “mental health staff” OR “healthcare provider*” OR “healthcare worker*” “healthcare professional*” OR “healthcare personnel” OR “healthcare staff” OR “clinic* staff” OR “clinic* personnel”

app OR application

mobile OR smartphone

“mental health” OR psych* OR wellbeing OR well-being OR “well being” OR wellness OR depression OR anxiety OR mood
