# Supplementary material for: Navigating the Digital Landscape for Potential Use of Mental Health Apps in Clinical Practice: Scoping Review
Source: JMIR Ment Health. 2026 Jan 15;13:e75640. doi: 10.2196/75640 (PMC12856407; doi:10.2196/75640)
Supplement: Multimedia Appendix 3 [file mental_v13i1e75640_app3.docx]

|  | **Study characteristics** | | **Characteristics of Practitioners** | | **Characteristics of Clients** | | | **App Characteristics** | | |
| --- | --- | --- | --- | --- | --- | --- | --- | --- | --- | --- |
| **Author (1^st^ only)** | **Aim** | **Primary Methodology and Design** | **Age ^[[1]](#footnote-1)^**  **Mean (SD)** where available | **Gender (%)** | **Age Mean (SD)** | **Mental Health Condition** | **Population** | **App Name** | **App Function** | **Patterns of Use** |
| Adams [50] | To develop and perform initial usability pilot-testing of a mobile app designed to augment outpatient behavioural health treatment for adolescents with substance use disorders and co-occurring mental health concerns | • Mixed methods (Pilot) • Semi structured interviews • Questionnaires | • Alpha: 43.3 (7.5) • Beta 2: 37.8 (12.1) | Female (100%) | • Alpha: 15.3 (1) • Beta: 15.9 (1) | Substance use disorders | Clinical | Bright Path | • Inform • Record - Collect • Instruct | Not reported |
| Almadani [51] | To investigate MH-App use among Saudi Arabian healthcare workers (HCWs) and assess their interest in and willingness to integrate these apps into their practices, as well as their cognitive flexibility and its relationship with their attitudes and app use | • Quantitative  • Questionnaires/ Survey  • Cross-sectional | <30 (61.1%) 30-40 (30.1%) 41-50 (06.0%) >50 (02.8%) | 53.40% | Not reported | Not reported but likely multiple conditions | Not Reported but likely Clinical | Recommended a variety of apps including Qariboon (31.1%), Calm (19.8%), Alseha Alnasfsia (19.8%), Headspace (14.2%) | • Inform  • Instruct • Record - Collect • Communicate | 27.5% of HCWs recommended mental health apps to their patients, 71.2% intended to use the apps in the future for patient-related purposes. |
| Anastasiadou [52] | To test the acceptability and feasibility of a mHealth tool for patients with an eating disorder among patients and specialists | • Qualitative • Focus groups | 34.63 (7.21) | Female (75%) | 15 (0.5) | Eating disorders | Clinical | TCApp | • Record - Collect, Share • Remind/Alert • Communicate | Not reported |
| Armstrong [53] | To explore youth and mental health provider perspectives on using apps to enhance evidence-based clinical care, with an emphasis on behaviour-tracking apps | • Qualitative • Semi structured interviews | 38.42 (5.78) | Female (67%) | 18.9 (3.73) | Not reported (General mental health) | Clinical | N/A | • Record - Collect | Not reported |
| Bucci [54] | To explore frontline staff views regarding the utility and appropriateness of using digital tools in the healthcare pathway for people accessing specialist secondary care mental health services | • Qualitative • Focus groups | 36.2 (SD not reported) | Female (56%) | Not reported | Psychosis | Not reported (but is likely clinical) | N/A | • Record - Collect, Share, Evaluate, Intervene | Not reported |
| Chang [55] | To conduct interviews with both patients and clinicians about their experience with visualisations being integrated into their care | • Qualitative  • Semi-structured interviews | Not reported | Not reported | 37 (10.7) | Depression  Anxiety | Clinical | mindLAMP | • Record - Collect • Remind/Alert  • Display | 2 clinicians did not often utilise the app visuals in clinical settings. 1 clinician found that the reports rarely, if ever, augmented patient care. |
| Cheung [56] | To identify the perceived facilitators and barriers to technology-enhanced measurement based care from both patient and provider perspectives | • Quantitative (cross-sectional) • Survey | Mode:  60+ (34.3%) | Female (40.7%) | • 18-29 (20.5%) • 30-39 (24.4%) • 40-49 (20.6%) • 50-59 (22.9%) • 60+ (13%) | Depression | Clinical | N/A | • Record - Collect, Share, Evaluate | Not reported |
| Deady [57] | To systematically detail the development process used to build a smartphone app that supports face-to-face clinical treatment for PTSD | • Mixed Methods  • Semi-structured interviews  • Questionnaires/Survey | Not reported | 100% | 50 (range 42-56) | PTSD Anxiety Depression | Clinical | Support Base | • Inform • Record - Collect, Share • Remind/Alert  • Communicate | Two clinicians stated they would be 'somewhat likely' to incorporate an app as a treatment support tool, while the other was 'neither likely nor unlikely'. |
| Dobson [58] | To explore the digital competence of psychologists and their use of digital tools in the practice | • Mixed methods • Survey | Mode:  24-35 (37%) | Female (88%) | Not reported | Not reported | Not reported | N/A | Not reported | Not reported |
| Dominiak [59] | To assess the attitudes, expectations, and concerns of mental health professionals towards mHealth, in particular mobile health self-management tools and telepsychiatry | • Mixed Methods  • Questionnaires/Survey | 25-39 (27%) 40-55 (56%) 55-64 (15%) | 67% | Not reported | Not reported | Not Reported | N/A | • Record - Collect | Between 2020 and 2023, 71.43% to 74.19% of participants would recommend apps to patients. |
| Dubad [60] | To explore the clinical impacts of mobile mood-monitoring in youth with mental health problems | • Mixed methods • Semi structured interviews | Not reported | Not reported | 20.71 (2.56) | • Psychotic disorders • Mood, panic, eating, and/or anxiety disorders | Clinical | Catch It | • Record - Collect | Not reported |
| Etingen [61] | To examine VHA mental health provider perspectives on the MHC app, including impacts that using the app has on mental health care delivery and outcomes. | • Mixed Methods  • Semi-structured interviews  • Questionnaires/Survey | Under 35 (19.2%) 36-45 (50.5%) 46â€“55 (19.2%) 56â€“65 (9.3%) 66-75 (1.6%) | 80.70% | Not reported | PTSD  Depression Anxiety Military sexual trauma Adjustment disorders/stress reactions Substance use disorder Bipolar disorder Psychotic disorders  Other diagnoses | Clinical | Mental Health Checkup (MHC) | • Record - Collect, Share • Remind/Alert  • Display | Most had assigned assessments through MHC to veterans, and had reviewed these assessments. |
| Francese [62] | To assess (i) the acceptability of an app that monitors mood by images, speech and questionnaire answers for use in clinics and (ii) the patient user experience. | • Quantitative • Questionnaire | 43 (6.18) | Not reported | 36.24 (14.02) | Depression | Clinical | EDApp | • Record - Collect, Share, Evaluate • Display | Not reported |
| Gonzalez-Perez [63] | A geolocation-based smartphone software component was designed and developed to instrumentalise patient guidance in in-vivo ET and its psychological validity was assessed | • Mixed Methods  • Questionnaires/Survey | 35 (SD not reported) | 66.60% | Not reported | Anxiety disorders | Not Reported but likely Clinical | SyMptOMS-ET | • Inform  • Record - Collect • Remind/Alert • Guide | Not reported |
| Green [64] | To understand the methods to implement and support technology in routine coordinated specialty care (CSC) and offered insights from a quality improvement study assessing the implementation outcomes of digital mental health interventions (DMHIs) in CSC. | • Mixed Methods  • Questionnaires/Survey | Not reported | Not reported | 19.6 (2.05) | Psychosis | Clinical | mindLAMP | • Inform • Instruct • Record - Collect, Share • Remind/Alert | Not reported? |
| Heydarian [65] | To identify the features of designing a mobile application for BP-affected patients | • Mixed Methods  • Semi-structured interviews  • Questionnaires/Survey | Not reported | Not reported | Not reported | Bipolar disorder | Clinical | N/A | • Inform  • Record - Collect, Share • Remind/Alert  • Display | Not reported |
| Hildebrand [66] | To explore psychotherapists’ perspectives on the barriers and facilitating conditions to internet- and mobile-based interventions. | • Qualitative  • Questionnaires/Survey  • Cross-sectional | 42.83 (12.16) | 76.90% | Not reported | Anxiety Depression Stress-related Substance abuse Pain | Clinical | N/A | • Inform • Record - Collect • Communicate | 26.29% reported already prescribing IMIs. |
| Hoffman [67] | To test the feasibility of using mental health applications to augment integrated primary care services; solicit feedback from patients and providers to guide implementation, and develop a mental health apps toolkit for system-wide dissemination | • Mixed methods (longitudinal pilot) • Survey | Not reported | Not reported | 36.5 | • Anxiety • Stress • Depression • Alcohol and tobacco use | Clinical | Variety - from Cambridge Health Alliance's mobile app toolkit including Breahe2Relax, Mindfulness Coach, Self-Help for Anxiety Management, CBT-iCoach, MoodTools, T2 MoodTracker, QuitforLife | • Record - Collect, Intervene • Inform • Guide | The majority (82.6%) incorporated apps into their clinical work |
| Kerst [68] | To explore the attitudes of health care professionals towards their use of apps for depression in practice | • Quantitative • Survey | 43 (12.3) | Female (64.9%) | Not reported | Depression | Not reported | N/A |  | 21.1% indicated that they used apps in clinical practice before |
| Khan [69] | To understand stakeholders’ opinions and experiences on how to choose a Mhealth App. | • Qualitative  • Semi-structured interviews | Range: 32-48 | Not reported | Range: 32-48 | Not reported but general mental health, and at least one affective disorder | Not Reported but likely Clinical | N/A | • Inform • Record - Collect • Remind/Alert | One clinician mentioned that they had not recommended any apps, while the other clinician noted that some patients successfully integrated apps into their daily routines and reported back as part of continued follow-up care. |
| Li [70] | To design a new CBT smartphone app that targets depressive and anxiety symptoms in adolescents | • Quantitative • Survey | 38.7 (10.4) | Female (81.3%) | 14.94 (1.3) | • Depression • Anxiety | • Clinical • Non-clinical | ClearlyMe | • Inform • Record - Collect Share | Not reported |
| Lukka [71] | To create a contextual understanding of how MHPs use different digital tools in clinical client practice and what characterises the use across tools | • Qualitative  • Semi-structured interviews | 18-29 (5%) 30-39 (16%) 40-49 (32%) 50-59 (37%) 60-69 (11%) | 68% | Not reported | Not reported | Clinical | N/A | • Inform (although unclear if used for other purposes also) | The MHPs rarely used or recommended mobile apps to their clients. |
| McGee-Vincent [72] | To develop, disseminate, and evaluate a training for multidisciplinary staff as part of a national quality improvement project to increase the reach of mobile mental health apps as a resource for veterans | • Quantitative  • Questionnaires/Survey | MHSL staff (44.2, 10.2) AOSL staff (46.0, 10.9) | MHSL staff (71.8%) AOSL staff (79.1%) | Not reported | Not reported | Clinical | N/A | Not Reported | 61.7% reported that they would definitely recommend the VA MH apps to veterans at baseline, increasing to 84.5% following training |
| Medich [73] | To analyse the acceptability and usability of passive mobile monitoring and self-tracking among patients with serious mental illness and their mental health clinicians | • Qualitative  • Semi-structured interviews  • Focus groups | Not reported | Not reported | Median: 45, Range: 21-66 years | Serious mental illness | Clinical | Mobile Sensing | • Record - Collect, Share • Remind/Alert  • Display | Most had no experience using mobile apps for mental health in their clinical practice, but said they would recommend the Mobile Sensing app to their patients. |
| Miller [74] | To evaluate the use and perceptions of apps among Primary Care Mental Health Integration (PCMHI) providers | • Mixed methods • Survey | 45.5 (11.1) | Female (78.6%) | Not reported | Apps were used primarily for insomnia or sleep disturbances (76.4%), stress (75.8%), PTSD (65.9%), depression (65.9%), and/or anxiety (64.3%). | Not reported | The most commonly used VA-created apps were Mindfulness Coach (67.6%), CBT-i Coach (56%), PTSD Coach (50.5) | • Inform • Record - Collect • Remind/Alert • Guide | Majority (82.7%) recommended or used apps with their patients |
| Morton [75] | To explore clinicians’ attitudes towards, and use of apps when working with people with bipolar disorder (BD) | • Mixed methods • Survey | 44.7 (13.1) | Female (53.8%) | Not reported | Bipolar disorder | Not reported | N/A | • Record - Collect • Inform | Approximately half (48.8%) discussed or recommend health apps with patients |
| Naccache [76] | To explore early acceptability and user experience of a companion app prototype for adolescents with anorexia nervosa (AN) using user-centred design methods | • Mixed methods • Semi structured focus group interviews • Questionnaires | 36.7 (7.38) | Female (57.1%) | 15.5 (1.07) | Anorexia nervosa | Clinical | Not reported | • Inform • Guide • Record - Collect, Evaluate • Remind | Not reported |
| Nogueira-Leite [77] | To understand the attitudes and expectations of psychiatrists and psychologists toward digital mental health apps (DMHAs) in the Portuguese context, as well as perceived benefits, barriers, and actions to support their adoption | • Mixed Methods  • Semi-structured interviews  • Questionnaires/Survey  • Cross-sectional | <26 (2.5%) 26-35 (35%) 36-45 (36.9%) 46-55 (19.4%) 56-65 (3.8%) >65 (2.5%) | 83.80% | Not reported | Not reported | Clinical | N/A | • Record - Collect | 42.5% declared to have an increased likelihood to prescribe DMHAs in the coming 12 months, although almost none had prescribed digital MHapps to their clients to date. |
| Orengo-Aguayo [78] | Assess providers’ views of the use of technology in the delivery of an empirically supported mental health treatment for adolescents (Trauma-Focused Cognitive Behavioral Therapy; TF-CBT) | • Mixed methods • Semi structured focus group interviews • Survey | • Phase 1: 41.88 (10.25) • Phase 2: 39.73 (10.26) | Not reported | Not reported (although we know that the clinicians treated children and adolescents) | Trauma-related conditions | Not reported (but is likely clinical) | N/A | • Remind/Alert • Record - Collect • Communicate • Inform | Not reported |
| Patoz [79] | To identify, through a user-centred design approach, patient and physician expectations of a hypothetical app dedicated to depression | • Qualitative • Semi-structured interviews | 45.5 (12.2) | Female (50%) | 51.5 (15.5) | Depression | Clinical | N/A | • Record - Collect, Share, Evaluate, Intervene • Display • Communicate • Inform • Guide • Remind/Alert • Instruct | Not reported |
| Puhy [80] | To investigate whether the mHealth software platform, NeuroFlow, was usable and acceptable for students receiving mental health services and for their treatment providers | • Mixed methods • Focus groups • Questionnaire | Not reported | Not reported | 16.1 (1.2) | Most common disorder was depression (44.4%, n = 4) | Clinica (5+ sessions) | NeuroFlow | • Remind/Alert • Record - Collect, Share, Evaluate, Intervene • Inform • Guide • Communicate • Display | Not reported |
| Richards [81] | To investigate therapist views on their experiences using a technological adjunct (goACT) to traditional, face-to-face psychotherapy | • Qualitative • Focus groups | Not reported | Not reported | Not reported (although we know that the patients were 18 years and older) | Not reported | Not reported (but is likely clinical) | goACT | • Communicate • Remind/Alert • Record - Collect, Share | Not reported |
| Rodriguez-Villa [82] | To engage clinicians, and people living with schizophrenia spectrum disorders and their family members in developing new features and co-designing the mindLAMP app and assess its effectiveness in predicting and preventing relapse | • Qualitative • Focus groups | • BIDMC: 41 (SD not reported) • AIIMS: 35 (SD not reported) • NIMHANS: 41 (SD not reported) | Not reported | • BIDMC: 32 (SD not reported) • AIIMS: 33 (SD not reported) • NIMHANS: 35 (SD not reported) | Schizophrenia | Clinical | mindLAMP | • Record - Collect, Evaluate  • Display • Guide • Remind/Alert • Inform • Instruct • Communicate | Most providers had suggested mindfulness apps to patients, and some  recommended apps focused on clinical outcomes such as DBT diary card or CBT apps. |
| Rothmann [83] | To develop and pilot test a telepsychology module for inclusion in the app My Hospital to provide remote psychological counselling to vulnerable adults with either rheumatic diseases or diabetes | • Qualitative • Semi-structured interviews | Not reported | Not reported | Only reported range = 25-59 years | Not reported | Referred to clinic due to psychological challenges related to living with chronic rheumatic disease or diabetes | My Hospital | • Inform | Not reported |
| Stefancic [84] | To describe the systematic process of soliciting inputs from clinician stakeholders to develop First Episode Digital Monitoring (FREEDoM), an app-based mHealth intervention | • Qualitative • Semi-structured interviews | Not reported | Not reported | Not reported (although we know the clinics provide treatment to adolescents and young adults aged 16-30 years) | Psychosis | Assume Clinical | First Episode Digital Monitoring (FREEDoM) | • Record - Collect, Share, Evaluate • Inform • Communicate • Instruct • Display | Not reported |
| Strodl [85] | To explore the perceptions of PTSD Coach Australia by clinicians with experience in assisting Defence members with mental health problems | • Mixed methods • Focus groups • Semi-structured interviews • Questionnaire | • Focus group: 47 (10.5) • Telephone sample: 45.7 (10.5) | • Focus group: Female (73%) • Telephone sample: Female (80%) | Not reported | PTSD/ Trauma | Not reported | PTSD Coach | • Inform • Record - Collect, Share  • Guide • Remind/Alert | Not reported |
| Weermeijer [86] | To evaluate the usability of an experience sampling method (ESM) protocol for using the ESM in a specialised mental health care setting | • Mixed Methods  • Semi-structured interviews  • Questionnaires/Survey  • Pilot | Users: 45.57 (6.11) Dropouts or nonusers: 43.50 (17.50) | Users: 88% Dropouts or nonusers: 25% | Users: 34.93 (11.27) Dropouts or nonusers: 36.67 (13.47) | Not reported (likely multiple including substance abuse, OCD, stress) | Clinical | IMPROVE | • Record – Collect, Share • Remind/Alert  • Display | Not reported |
| Wu [87] | To understand clinicians’ perspectives on patient-generated health data (PGHD) and current mental health apps | • Mixed methods • Semi structured interviews | Not reported | Female (41.6%) | Not reported | Not reported | Not reported | 31 sleep and mood tracking apps | • Record - Collect, Share, Evaluate • Display | Not reported |

1. *Mean age and gender typically reported for all practitioners, and sometimes all of sample.* [↑](#footnote-ref-1)
